# Supplementary material for: Modulations in martensitic Heusler alloys originate from nanotwin ordering
Source: Sci Rep. 2018 May 31;8:8489. doi: 10.1038/s41598-018-26652-6 (PMC5981613; doi:10.1038/s41598-018-26652-6)
Supplement: Supplementary file 1 — Supplementary information [file 41598_2018_26652_MOESM1_ESM.pdf]

Supplementary information for

Modulations in martensitic Heusler alloys originate from nanotwin ordering

M. E. Gruner[1,2], R. Niemann[2], P. Entel[1], R. Pentcheva[1], U. K. Rößler[2], K. Nielsch[2], S. Fähler[2 *]

[1] *Theoretical Physics and Centre for Nanointegration, CENIDE, University of Duisburg-Essen, D-47048 Duisburg, Germany*

[2] *IFW Dresden, Helmholtzstraße 20, D-01069 Dresden, Germany*

corresponding author's email: S.Faehler@ifw-dresden.de

This supplementary information is organised in the same order as the paper. The supplementary figures are numbered from S1 to S6.

To Section 2: Oscillating interaction energy of nanotwins in dependency of their spacing

Energy of ideal nanotwins along the Bain path

In order to understand the influence of the tetragonal distortion on the interaction energy, we expanded our calculation for the ideal nanotwinned arrangement to values below 1.26. Fig. S1 shows the evolution of the twin boundary energy of unrelaxed symmetric twins as a function of the twin width for different tetragonal distortions $c/a|_{\mathrm{NM}}$ of the NM building blocks. The total twin boundary energy, γ_{tot}, was calculated from the energy difference between the twinned structure, E_{twin}, constructed for a given $c/a|_{\mathrm{NM}}$ and the respective non-modulated building block, E_{NM}, using the relation:

$$\gamma_{\mathrm{tot}}(c/a|_{\mathrm{NM}}) = \frac{E_{twin}(c/a|_{\mathrm{NM}}) - E_{\mathrm{NM}}(c/a|_{\mathrm{NM}})}{2\,A_{\mathrm{base}}}\;(n+m)$$

The numerator denoted the energy difference per formula unit. n and m described the number of twins with the respective orientations (i.e., $n=m$ in the symmetric case) and A_{base} denoted the area of the base plane spanned by the short lattice vectors a and b of the unit cell of the twinned structure. The factor 2 was present in the denominator because twin interfaces always occur pairwise according to the periodic boundary conditions used in the calculations.

The red curve for $c/a|_{NM} = 1.26$ in Fig. S1 corresponds to the result presented in Fig. 2 of the main manuscript. Close to the equilibrium distortion of the NM structure, the decrease of $c/a|_{NM}$ was found to have only little effect on the interaction part, while the interface part becomes effectively negative. This could be seen a consequence of the construction of the twin configuration from non-equilibrium building blocks, which describe intermediate states along the Bain path. Based on this, we concluded that with an additional shuffle at the twin interfaces, the system could approach the ground state more efficiently than in a transformation along the Bain path. At $c/a|_{NM} = 1.11$ and below, the interaction pattern changed and twin widths of $n = 5$ and $n = 3$ became first competitive with $n = 2$ and then preferred for decreasing values of $c/a|_{NM}$. In part, we interpreted this as a consequence of the electronic instability at the Fermi surface, which favours modulations with a periodicity of around six lattice planes. Because this contribution also enters into the interaction part, the additive relation $\gamma_{tot} = \gamma_{\infty} + \gamma_n$ strictly did not hold anymore for $c/a|_{NM} < 1.1$ where the NM building blocks exhibited imaginary phonon frequencies.

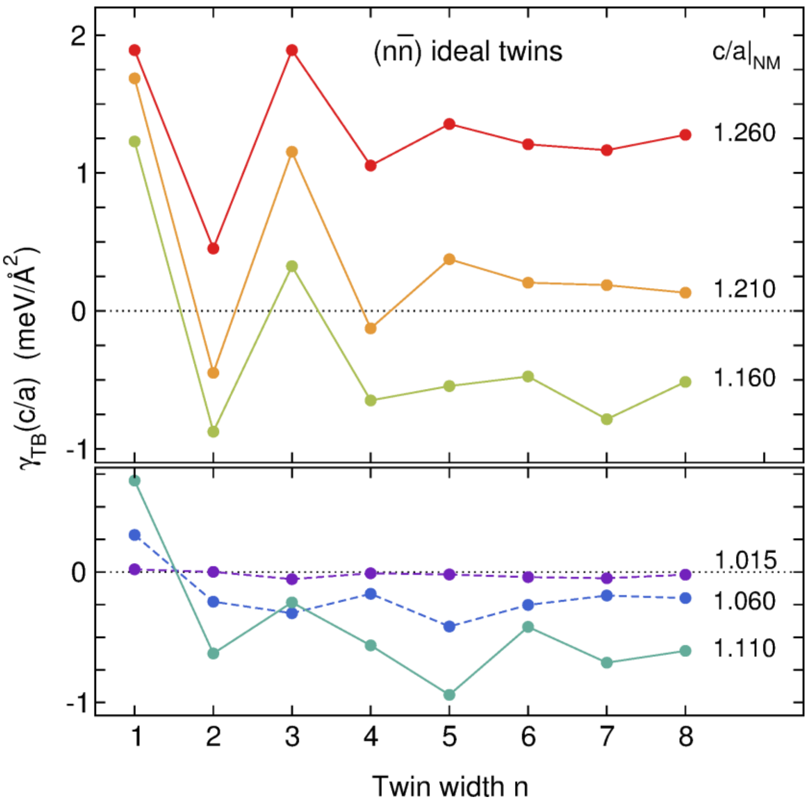

Fig. S1: Twin boundary energy of ideal unrelaxed symmetric $(n\bar{n})$ nanotwinned arrangements as a function of the twin width *n* for different tetragonal distortions $c/a|_{NM}$. The red curve (top in the upper panel) represent the results for $c/a|_{NM} = 1.26$ already shown in Fig. 2 of the main manuscript.

To Section 4: Ordering contributed to hysteresis losses

Calculation of the energy dissipated during ordering

To analyse the energy dissipated during ordering, we numerically calculated the total interaction energy of a random arrangement $(n_1, m_1, \dots, n_i, m_i \dots)$ of 10^9 twin variants with sizes between 1 and 9. The random list was constructed under the boundary condition of adaptivity:

$$\sum_i (m_i a_{\text{NM}} + n_i c_{\text{NM}}) = \sum_i (m_i + n_i) a_{\text{A}}$$

The total interaction energy of this disordered arrangement was given by

$$E_{\text{disordered}} = \frac{1}{L} \sum m_i \gamma_{\text{TB}}(m_i) + n_i \gamma_{\text{TB}}(n_i)$$

L was the total length of the model crystal. The energies γ_{TB} for the relaxed case were used (Fig. 2). The interaction energy of a disordered arrangement then amounted to 4.1 MJ/m^3. An ordered arrangement of the same number of unit cells would only consist of twins with $n = 5$ and $m = 2$ (for 14M). Hence, the total interaction energy of this perfectly ordered arrangement was given by

$$E_{\text{disordered}} = \frac{1}{L} \sum \frac{m_i}{5} \gamma_{TB}(5) + \frac{n_i}{2} \gamma_{TB}(2)$$

The result was approximately 0.2 MJ m^{-3}, which means that the energy dissipated during ordering was 3.9 MJ m^{-3}.

To Section 4: Magnetic origin of the interaction energy

Magnetic perturbations at the twin interface

Fig. S2 demonstrated the close interrelation of the oscillatory twin boundary interaction with the perturbation of the magnetic subsystem at the interface in terms of the magnetisation profile of the local moments associated with the Ni-atoms perpendicular to the twinning plane (vertical line at zero in Fig. S2).

In Ni_2MnGa, the Mn-moments can be considered as predominately localised because they do not significantly change their magnitude within a spin-spiral, antiferromagnetic or paramagnetic arrangement. In turn, the magnetic moments associated with the Ni-atoms are

essentially itinerant and induced by the interaction with the surrounding Mn-Moments.[1] This relation is expressed in the large nearest neighbour Mn-Ni exchange constant, which decays very fast and has essentially vanished for next-nearest neighbours. Thus, one can expect that charge neutral perturbations of the electronic subsystem arising from the interface will be visible in the effective spin-polarisation of the itinerant Ni electrons.

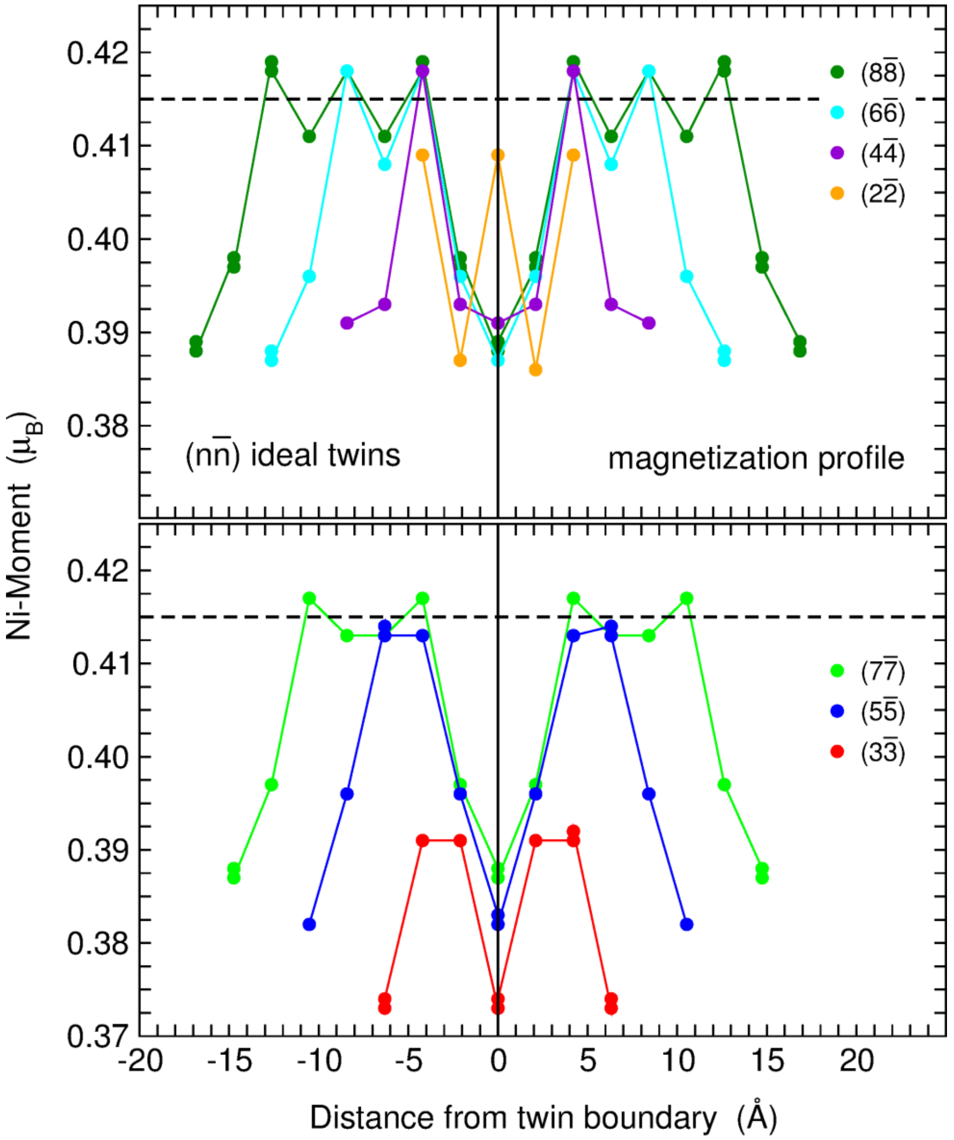

Fig. S2: Magnetisation profiles from DFT calculations of the local magnetic moments of the Ni-atoms in the symmetric ideal (unrelaxed) twin structures. The ordinate describes the distance of the respective atom from the central twin boundary (black vertical line at zero). The outermost data points correspond to the next-nearest twin boundary planes. The lower panel shows the structures with an odd number of twin layers, the upper panel those with even layered twins. The dotted lines indicate the equilibrium Ni-moment obtained for the corresponding untwinned NM.

In large twins with a width of five layers and above, the moments of the Ni atoms fluctuated around the equilibrium value observed in the NM bulk structure (dashed horizontal line). This suggested that these atoms, located in sufficient distance from the interface, did not encounter a significant perturbation. In contrast, the Ni-atoms located directly in the twin boundary exhibited a spin polarization that was reduced by about 7%. The adjacent layers were also affected by a reduction in spin polarisation of 5%. For the well-localised magnetic moments of Mn atoms, the oscillation was much smaller, typically around 0.01 μ_B in the vicinity of well separated interfaces, which corresponds to 0.3%. Consequently, if we decreased the number of layers in one twin to four layers – where in Fig. 2 of the main manuscript the oscillatory twin interaction becomes important – the perturbed regions first come into contact, and finally interpenetrated and superimposed for smaller twins. Eventually, also the spin-polarisation of Ni at the interface was affected. This had different consequences for even and for odd numbered twins. In the first case, the magnetic moments of Ni at the twin boundary increased with decreasing width *n*, whereas they further decreased in the latter case.

To Section 6: Twin boundary energy determined the phase sequence from A→10M→14M→NM(4O)

Energies of unrelaxed modulations

For completeness, we present the energies of unrelaxed, ideal twin structures in Fig. S3 in addition to the structures shown in Fig. 6 of the main manuscript that underwent a full relaxation with respect to their atomic positions. The energy of an ideal twin was found to be somewhat higher compared to its optimised counterpart. Nevertheless, all nanotwinned structures were found to be lower in energy than the NM structure in the range $1.0 < c/a|_{NM} < 1.2$, with an exception for the $(2\bar{1})_2$ modulation. At $c/a|_{NM} < 1.05$, all nanotwins were observed to be energetically degenerate with austenite, except for the $(2\bar{3})_2$ modulation**,** which decreased with increasing $c/a|_{NM}$. We ascribed this again to its favourable modulation period that can benefit from Fermi-surface nesting. Because it was lower in energy (except for the end point), nanotwinning could offer an improved alternative to the Bain transformation path from austenite to tetragonally distorted martensite, even without further shuffling of the

atomic positions. Relaxation opened the possibility to gain additional energy from the soft phonon at small $c/a|_{NM}$ and from the lifting of interface stresses at large $c/a|_{NM}$. We expected both of these aspects to be central factors in the formation of modulated martensites, considering the newly found transformation path.

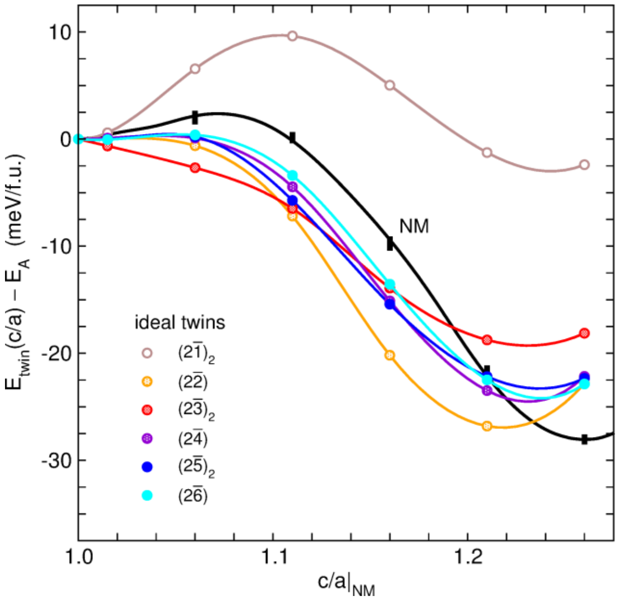

Fig. S3: Energy difference of ideal unrelaxed asymmetric $\left(n\bar{m}\right)_2$ nanotwinned arrangements with respect to the cubic austenite as a function of the tetragonal distortion $c/a|_{NM}$. The black line denotes the energy of a non-modulated tetragonal unit cell.

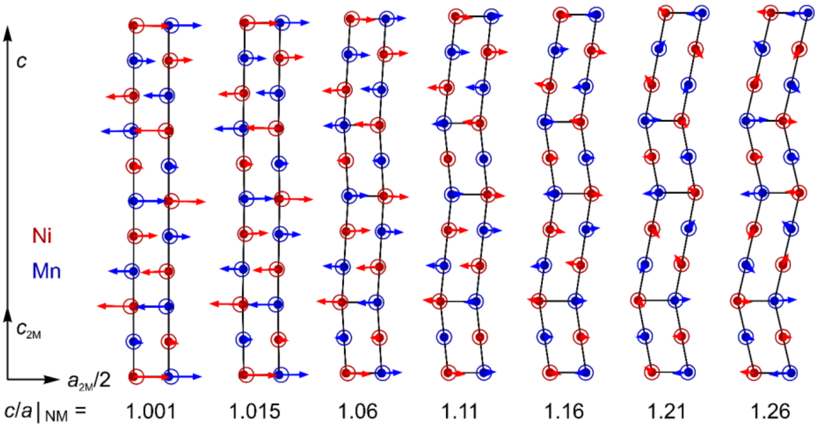

Fig. S4: Relaxation of 10M with increasing tetragonal distortion $c/a|_{NM}$. The positions of the atoms in a nanotwinned arrangement are depicted by solid circles and the relaxed positions by open circles. The Ga atoms underwent a similar relaxation compared to Mn and are thus omitted. The arrows, illustrating the direction and magnitude of the movement of the atoms, were enlarged by a factor of five for better

visibility. This projection used the smallest unit cell of modulated martensite (2M) in contrast to the $L2_1$ description in Fig. 1.

To Section 7: Soft phonons facilitated the transition to a nanotwinned martensite

Relaxation of 10M

In addition to the image of the lattice in real space of 14M in Fig. 6, we show the 10M lattice in Fig. S4. We did not find a qualitative difference to 14M; in particular, we observed a strong tendency to relax at low $c/a|_{NM}$, while the equilibrium positions were almost identical to the positions predicted by the adaptive concept at higher $c/a|_{NM}$.

Fourier analysis of the modulation

Here, we describe the calculation of the Fourier coefficients presented in Fig. 7 for 14M and in Fig. S5 for 10M. To analyse the character of the modulation, a discrete Fourier analysis of the modulation amplitude was carried out. First, the distance of the atoms perpendicular to the *c*-axis was calculated (Fig. S4). For the relaxed case, we found that the *x*-position of the atoms along the *c*-axis were not equally distributed. Therefore, the amplitude was interpolated using a third order polynomial to an equally distributed list of amplitudes before applying the Discrete Sine Transform (DST-II):

$$v_s = \frac{1}{\sqrt{n}} \sum_{r=1}^{n} u_r \sin\left(\frac{\pi}{n}\left(r - \frac{1}{2}\right) s\right)$$

The v_s were the coefficients for the different order s of the Sine transform. The modulation function were synthesised using:

$$y(x) = \sum v_s \sin\frac{2\pi}{\lambda_s}\left(x + \frac{1}{2}\right)$$

where $\lambda_s = c/s$. For simplicity, λ_s is shown in the figures instead of s. Fig. S5 shows an example of a Fourier synthesis for the relaxed case of 14M at $c/a|_{NM}$ = 1.26 in real space. The *x*-axis was parallel to *c*, while the y-values were the perpendicular distances of the atoms to the *x*-axis (amplitudes). If only $\lambda_2 = c/2$ was taken into account for the synthesis, the modulation was found to be only poorly described by a single sinusoidal function. If one takes into account all the terms with *c*/2, *c*/4 and *c*/6, the description was much better, meaning

that, in this case, the modulation was not sinusoidal. In general, the more terms above λ_2 were different from zero, the less sinusoidal and smooth was the modulation. The coefficients for 10M are shown as a function of the order in Fig. S6, in addition to the results for 14M presented in Fig. 6.

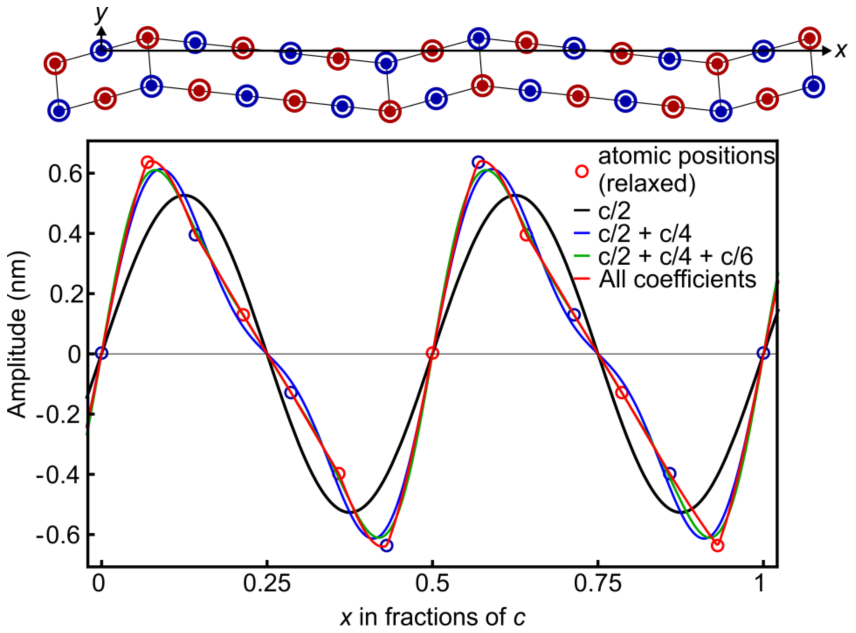

Fig. S5: Fourier synthesis of 14M modulations at $c/a|_{NM}$ = 1.26 in comparision with the relaxed atomic positions. Top: Lattice in real space of the relaxed positions (open circles), and of an ideal 14M nanotwinned martensite (closed circles). The long axis is parallel to *x*, the short axis is perpendicular to *x* and describes the distance of the atoms from the *c*-axis, which is the amplitude of the modulation. Bottom: Relaxed atomic positions (circles) in comparison with different Fourier syntheses. *c*/2 describes a pure sine function; the other functions are of higher order as indicated.

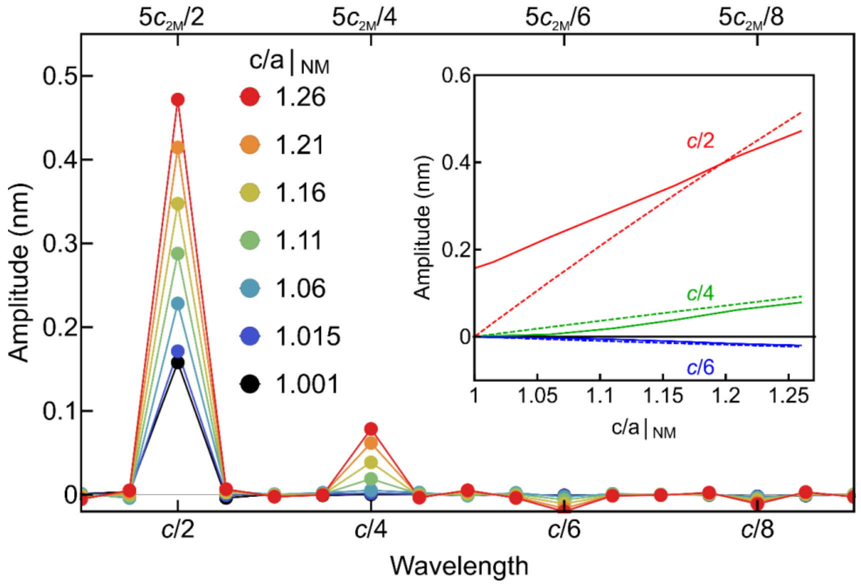

Fig. S6: Quantifying the transition from a sinosoidal phonon instability to a zig-zag-like nanotwinning in 10M. Discrete sine Fourier transformation coefficients of the relaxed lattice position in 10M, describing the average movement of all atoms within one plane perpendicular to the modulation vector *c*. While the bottom axis gives the wavelength with respect to the full 2M-based unit cell of modulated martensite (long *c*-axis), the top one uses the reduced 2M description (compare to Fig. S4). The inset summarises the main components in dependence of $c/a|_{NM}$ for the relaxed atoms (solid lines) in comparison with the unrelaxed atoms (dashed lines). In the relaxed case and for low tetragonal distortions, only the first harmonic was required, while for large distortions, more components were required to describe the zig-zag arrangement of the nanotwin boundaries correctly.

Reference

[1] Enkovaara, J., Ayuela, A., Jalkanen, J., Nordström, L., Nieminen, R. M. First-principles calculations of spin spirals in Ni_2MnGa and Ni_2MnAl, *Phys. Rev. B* **67**, 544171 (2003).
